# Astroglial PGC-1alpha increases mitochondrial antioxidant capacity and suppresses inflammation: implications for multiple sclerosis

**DOI:** 10.1186/s40478-014-0170-2

**Published:** 2014-12-10

**Authors:** Philip G Nijland, Maarten E Witte, Bert van het Hof, Susanne van der Pol, Jan Bauer, Hans Lassmann, Paul van der Valk, Helga E de Vries, Jack van Horssen

**Affiliations:** Department of Pathology, MS Center Amsterdam, VU University Medical Center, Amsterdam, The Netherlands; Department of Molecular Cell Biology and Immunology, Neuroscience Campus Amsterdam, VU University Medical Center, Amsterdam, The Netherlands; Department of Neuroimmunology, Center for Brain Research, Medical University of Vienna, Wien, Austria; Institute of Clinical Neuroimmunology, Ludwig-Maximilians University Munich, Munich, Germany

**Keywords:** Reactive astrocytes, Neurodegeneration, Prx3, Trx2, ROS

## Abstract

Recent evidence suggests that reactive oxygen species (ROS) produced by inflammatory cells drive axonal degeneration in active multiple sclerosis (MS) lesions by inducing mitochondrial dysfunction. Mitochondria are endowed with a variety of antioxidant enzymes, including peroxiredoxin-3 and thioredoxin-2, which are involved in limiting ROS-induced damage. In this study, we explored the distribution and role of the mitochondrial antioxidants peroxiredoxin-3 and thioredoxin-2 as well as their regulator peroxisome proliferator-activated receptor gamma coactivator1-alpha (PGC-1α) in MS pathogenesis.

Immunohistochemical analysis of a large cohort of MS patients revealed a striking upregulation of PGC-1α and downstream mitochondrial antioxidants in active demyelinating MS lesions. Enhanced expression was predominantly observed in reactive astrocytes. To elucidate the functional role of astrocytic PGC-1α in MS pathology, we generated human primary astrocytes that genetically overexpressed PGC-1α. Upon an oxidative insult, these cells were shown to produce less ROS and were found to be more resistant to ROS-induced cell death compared to control cells. Intriguingly, also neuronal cells co-cultured with PGC-1α-overexpressing astrocytes were protected against an exogenous oxidative attack compared to neuronal cells co-cultured with control astrocytes. Finally, enhanced astrocytic PGC-1α levels markedly reduced the production and secretion of the pro-inflammatory mediators interleukin-6 and chemokine (C-C motif) ligand 2. Our findings suggest that increased astrocytic PGC-1α in active MS lesions might initially function as an endogenous protective mechanism to dampen oxidative damage and inflammation thereby reducing neurodegeneration. Activation of PGC-1α therefore represents a promising therapeutic strategy to improve mitochondrial function and repress inflammation.

**Electronic supplementary material:**

The online version of this article (doi:10.1186/s40478-014-0170-2) contains supplementary material, which is available to authorized users.

## Introduction

Multiple sclerosis (MS) is the leading cause of non-traumatic neurological disability among young adults in Europe and North-America [1]. MS is generally characterized as an immune-mediated disease in which infiltrating macrophages and T-lymphocytes induce focal demyelination and neurodegeneration by producing large amounts of inflammatory cytokines and reactive oxygen species (ROS) [2,3]. In the process of inflammation-driven demyelination, astrocytes become activated and data is emerging that reactive astrocytes play an important and dual role in various processes underlying MS pathogenesis (for review see [4,5]). Reactive astrocytes can aggravate inflammation and blood–brain barrier (BBB) leakage by secreting inflammatory molecules, but also facilitate BBB repair, secrete immunosuppressive molecules and possess neuroprotective properties [6-9]. Importantly, astrocytes are the main source of antioxidants and are thus essential for scavenging ROS in MS lesions [10,11].

Macrophages and activated microglia produce significant amounts of ROS, which leads to increased mitochondrial ROS production thereby enhancing local oxidative stress and mitochondrial dysfunction [12]. ROS-mediated mitochondrial dysfunction is an important cause for axonal and neuronal degeneration, which are the pathological substrates of permanent disability in MS patients [13-15]. Mitochondria continuously produce ROS and are therefore equipped with a specific and efficient antioxidant apparatus, which under physiological circumstances efficiently controls the mitochondrial redox balance, thereby ensuring adequate mitochondrial function [16,17]. Peroxiredoxin-3 (Prx3) is the mitochondria-specific member of the peroxiredoxin family and catalyzes the reduction of various peroxides, a reaction in which peroxiredoxins are oxidized [18]. To regain its antioxidant capacity, Prx3 is reduced by mitochondrial thioredoxin-2 (Trx2), which by itself is also capable of directly reducing various ROS and oxidized proteins [19]. Proper function of Prx3 and Trx2 are of particular importance in the central nervous system (CNS) as neurons are highly dependent on a healthy mitochondrial population. In fact, different studies have indicated that Prx3 and Trx2 have neuroprotective properties [20,21].

Recently, we showed that the expression of mitochondrial antioxidants is reduced in the cortex of MS patients compared to control grey matter. Notably, the observed decrease strongly correlated with a significant decrease in the levels of transcriptional co-regulator peroxisome proliferator-activated receptor gamma co-activator 1-alpha (PGC-1α) [22]. PGC-1α has multiple binding partners and is thereby able to simultaneously induce transcription of a broad set of genes, most of which are involved in energy metabolism and redox handling, including Prx3 and Trx2 [23,24]. Thus far, data on the expression of PGC-1α and mitochondrial antioxidant enzymes in various stages of MS white matter lesions is limited. However, oxidative stress and mitochondrial dysfunction are most prominent in white matter lesions and contribute to axonal loss [25]. Therefore, it is imperative to understand and identify potential protective mechanisms aimed at restoring the redox balance and improving mitochondrial function in MS lesions. Hereto, we set out to explore the cellular distribution of key mitochondrial antioxidants and their transcriptional regulator PGC-1α in a large set of well-characterized MS white matter lesions.

We here describe that the expression of mitochondrial antioxidants and PGC-1α is markedly increased in inflammatory white matter lesions, particularly in reactive astrocytes. Overexpression of PGC-1α in human astrocytes reduced astrocytic ROS production and protects astrocytes from exogenous ROS-induced cell death. Moreover, neurons co-cultured with PGC-1α-overexpressing astrocytes are more resistant to exogenous ROS. Interestingly, PGC-1α also reduced interleukin-6 (IL-6) and chemokine (C-C motif) ligand 2 (CCL2) production by human astrocytes under normal and inflammatory conditions. Our data indicate that increased expression of PGC-1α and downstream mitochondrial antioxidants in astrocytes in MS lesions represents an intrinsic defense mechanism to restore the redox balance, suppress inflammation and promote neuro-axonal survival during an inflammatory-driven oxidative attack.

## Material and methods

### Brain tissue

Formalin-fixed, paraffin-embedded brain sections were obtained from 19 patients and 10 matched non-neurological controls from the Netherlands Brain Bank, Amsterdam and the Medical University Vienna, Austria. Detailed clinical data are summarized in Table 1. The study was approved by the institutional ethics review board (VU University Medical Center, Amsterdam; Medical University of Vienna EK Nr. 535/2004) and all donors or their next of kin provided written informed consent for brain autopsy, use of material and clinical information for research purposes.

**Table 1:** **Clinical data of MS patients and non-neurological controls**

**Case**	**Age (years)**	**MS type**	**Sex**	**Post-mortem delay (h:min)**	**Disease duration (years)**	**Lesion stages**
MS 1	73	ND	m	6:45	26	CIA
MS 2	63	PP	m	7:05	25	CA
MS 3	56	SP	m	8:00	27	CIA
MS 4	66	ND	m	7:45	ND	A
MS 5	41	PP	m	7:20	14	A, CA
MS 6	49	SP	m	8:00	25	CIA
MS 7	66	PP	m	7:30	26	2*CA
MS 8	61	SP	m	9:15	30	3*CA, CIA
MS 9	44	PP	m	12:00	13	CA
MS 10	44	SP	m	10:15	22	CA
MS 11	54	PP	m	8:15	15	3*A
MS 12	45	AMS	m	ND	1 week	3*A, CA, CIA
MS 13	35	AMS	m	ND	6 weeks	5*A, 2*CIA
MS 14	40	RR	f	ND	10	A
MS 15	34	AMS	f	ND	0,3	A
MS 16	78	AMS	m	ND	0,2	A, CA
MS 17	41	SP	m	ND	10,6	A
MS 18	46	SP	f	ND	37	CA, CIA
MS 19	51	AMS	f	ND	0,5	A, 3*CA
Ctrl 1	66	NA	f	7:00	NA	NA
Ctrl 2	71	NA	m	8:55	NA	NA
Ctrl 3	58	NA	m	5:15	NA	NA
Ctrl 4	62	NA	m	7:20	NA	NA
Ctrl 5	78	NA	m	17:40	NA	NA
Ctrl 6	51	NA	f	5:36	NA	NA
Ctrl 7	70	NA	m	ND	NA	NA
Ctrl 8	46	NA	m	ND	NA	NA
Ctrl 9	37	NA	m	ND	NA	NA
Ctrl 10	39	NA	f	ND	NA	NA

SP = secondary progressive MS; PP = primary progressive MS; AMS = Acute MS; ND = not determined; NA = non applicable; m = male; f = female; A = active lesion; CA = chronic active lesion; CIA = chronic inactive lesion.

### Immunohistochemistry

Five μm-thick paraffin sections were collected on Superfrost Plus glass slides (VWR international; Leuven, Belgium) and dried overnight at 37°C. Sections were pretreated and stained as described previously [26]. In short, sections were deparaffinized in a series of xylene (3 × 5 min), 100% ethanol, 96% ethanol, 70% ethanol and water. Endogenous peroxidase activity was blocked by incubating the sections in methanol with 0.3% H_2_O_2_. Next sections were incubated with appropriate primary antibodies (see Additional file 1: Table S1) in phosphate buffered saline (PBS) supplemented with 1% bovine serum albumin (BSA; Roche diagnostics GmbH, Mannheim, Germany) overnight at 4°C and stained with the EnVision horseradish peroxidase (DAKO, Glostrup, Denmark) kit followed by 3,3′diaminobenzidine-tetrahydrochloridedihydrate (DAB; DAKO). After a short rinse in tap water, sections were counterstained with haematoxylin for 1 min and intensely washed with tap water for 5 min.

Fluorescence immunohistochemistry in early active MS lesions was performed on paraffin sections as described previously [27]. For confocal fluorescent double labelling or triple labelling with primary antibodies from different species, antibodies were applied simultaneously at 4°C overnight (Additional file 1: Table S1). After washing with Dako washing buffer (DakoCytomation, Glostrup, Denmark), secondary antibodies consisting of donkey-anti-mouse Cy3 (Jackson ImmunoResearch, 1:200), biotinylated donkey-anti-rabbit (Amersham Pharmacia Biotech; 1:200) and Cy5-conjugated donkey-anti-goat, were applied simultaneously for 1 hour at room temperature, followed by application of streptavidin-Cy2 (Jackson ImmunoResearch; 1:75) for 1 hour at room temperature. Fluorescent preparations were embedded and examined using a confocal laser scan microscope (Leica SP5, Leica Mannheim, Germany) equipped with lasers for 504, 488, 543 and 633 nm excitation. Scanning for Cy2 (488 nm), Cy3 and Cy5 was performed sequentially to rule out fluorescence bleed through.

For colocalization studies in late active lesions, deparaffinized sections were incubated for 30 minutes with 10% animal serum, of which the source was determined by the specific secondary antibody used followed by incubation with the primary antibodies (Additional file 1: Table S1). Alexa Fluor® (Life Technologies, Vienna, Austria) labeled secondary antibodies were used for fluorescent labeling. Images were taken on a Leica DM6000 microscope (Leica Microsystems Heidelberg GmbH, Mannheim, Germany). All primary antibodies were diluted in 0.01 mol/L phosphate buffered saline (PBS; pH 7.4) containing 1% BSA and 0,05% Tween-20 (SigmaAldrich, StLouis, MO, USA), which also served as a negative control.

### Cell culture and lentiviral-induced (over)expression

Primary human cerebellar astrocytes (ScienCell, Carlsbad, CA) were cultured in astrocyte medium (ScienCell). The human neuroblastoma cell line SH-SY5Y and the human astrocytoma cell line U373 were both cultured in DMEM/F12 (1:1, Life Technologies) containing 10% foetal calf serum (FCS, Life Technologies), 2 mM L-glutamin (Life Technologies), and penicillin/streptomycin (50 mg/ml; Life Technologies) in 5% CO_2_ at 37°C.

To overexpress Prx3, its coding sequence was amplified from primary human astrocyte cDNA with primers (forward: CGGATCCCGATGGCGGCTGCTGTAGGA reverse: CCGAATTCCTACTGATTTACCTTCTGAAAG) and cloned into the lentiviral vector pRRL-cPPT-CMV-X2-PRE-SIN (kindly provided by Dr. J. Seppen, Academic Medical Center, Amsterdam, the Netherlands). The human Trx2 plasmid was kindly provided by Professor Jones (Emory University, Atlanta, GA, USA) and the human PGC-1α plasmid by Professor Strömstedt (Department of Pharmaceutical Biosciences, University of Oslo, Norway). Both vectors were amplified and cloned into the pRRL lentiviral vector [28,29]. Lentiviral vectors were produced by co-transfecting subconfluent human embryonic kidney (HEK) 293 T cells with the Prx3, Trx2 or PGC-1α expression plasmid and lentiviral packaging plasmids (pMDLg/pRRE and pRSV-Rev), using calcium phosphate as a transfection reagent. Lentiviral vectors were collected 24 h after transfection. The supernatant was centrifuged to remove cell debris and stored at −80°C. Human astrocytes were transduced with the lentivirus-containing PGC-1α. Human astrocytes overexpressing PGC-1α are indicated as PGC-1α^+^ astrocytes throughout the manuscript. U373 astrocyte-like cells were also transduced with PGC-1α and with Prx3 or Trx3 containing lentivirus since primary human astrocytes were scarce. Forty-eight hours after transduction, stable cell lines were selected by puromycin treatment (2 μg/mL; SigmaAldrich). The overexpression efficiency was determined by qPCR and western blotting and stable cell lines were used for functional studies described hereafter. Human astrocytes and U373 cells stably transduced with the empty pRRL vector served as control. Finally, SH-SY5Y neuroblastoma cells and human astrocytes were stably transduced with the SHC003 turboGFP vector (Sigma Aldrich), to generate a green fluorescent cell line.

### Real-time quantitative PCR

Total RNA from astrocyte cultures was isolated using Trizol (Invitrogen, Carlsbad, CA, USA) according to manufacturer’s protocol. mRNA concentrations were measured using Nanodrop (Nanodrop Technologies, Wilmington, DE, USA). cDNA was synthesized with the Reverse Transcription System kit (Promega, Madison, WI, USA) following manufacturer’s guidelines. Quantitative PCR (qPCR) reactions were performed in an ABI7900HT sequence detection system using the SYBR Green method (Applied Biosystems, Foster City, CA, USA) as described previously [30]. Obtained mRNA expression levels were normalized to XPNPEP1 (Qiagen, Venlo, the Netherlands) expression levels, which a recent study found to be the housekeeping gene of choice in human CNS studies and was in our hands also the most consistent in human primary astrocytes [31]. All oligonucleotides were synthesized by Ocimum Biosolutions (Ocimum Biosolutions, IJsselstein, the Netherlands) (Additional file 2: Table S2).

### Western blot

Protein isolation from astrocytes was performed using M-PER buffer supplemented with protease and phosphatase inhibitors according to manufacturer’s protocol (Thermo Scientific, Rockford, IL, USA). Protein concentrations were measured using BCA protein assay (Thermo Scientific). Western blot was performed as described previously [32]. In short, equal amounts of protein (25-100 μg) were separated on 10% SDS-PAGE gels and transferred to PVDF membranes (Bio-Rad Laboratories, Berkeley, CA, USA). After blocking in Odyssey blocking buffer (LI-COR Biosciences, Lincoln, AKUSA), membranes were incubated with appropriate primary antibodies (for details, see Additional file 1: Table S1) overnight in Odyssey blocking buffer at 4°C. Primary antibodies were detected by incubation with appropriate IRDye secondary antibodies (LI-COR Biosciences) for 1 hour at RT in Odyssey blocking buffer and quantified using the Odyssey infrared imaging system (LI-COR Biosciences).

### Functional analysis of cells overexpressing Prx3, Trx2 and PGC-1α

Stable cell lines transduced with Prx3, Trx2 and PGC-1α containing lentiviral vector or empty pRRL vector were plated in 96-well plates (Greiner Bio-One, Frickenhausen, Germany) and grown until confluent. Cells were treated with 200 μM of tbH_2_O_2_ for 6 hours. Hereafter, cell viability was assessed using the LIVE/DEAD Viability/Cytotoxicity kit (Invitrogen) according to the manufacturer’s protocol. Fluorescent signals were measured with the Fluostar Galaxy (BMG Labtech, Ortenberg, Germany) fluometer and the ratio between dead and live cells was calculated. Endogenous ROS production was assessed using 5-(and-6)-chloromethyl-2′,7′-dichlorodihydrofluorescein di-acetate, acetyl ester (CM-H2DCFDA; Invitrogen), a probe which turns fluorescent upon oxidation. Total fluorescence was measured with the Fluostar Galaxy (BMG Labtech, Ortenberg, Germany) and corrected for live cell number.

#### Enzyme linked immunosorbent assays (ELISA)

Secretomes of mock and PGC1α + astrocytes treated for 24 hours with or without TNF-α and IFN-γ (5 ng/ml; Peprotech, UK) were harvested and stored at −80°C for further analysis. The Human IL-6 Cytoset™ (Invitrogen) and Human CCL2 DuoSet® (R&D system, Minneapolis, MN) ELISA kits were used to determine the amount of IL-6 and CCL2 secreted by primary human astrocytes. ELISA was performed according to manufactures protocol and absorbance was analyzed at 450 nm with 570 nm wavelength correction on a spectrophotometer (Bio-Rad, Hercules, CA).

#### Statistical analysis

Student’s t-test was applied to determine differences between PGC-1α- and mock transduced astrocytes with regard to viability, ROS production and cytokine production. 2-Way ANOVA with Bonferroni post-hoc test was used to analyze the co-culture experiments and asses differences between Prx3-, Trx2- and mock-transduced astrocytes.

## Results

### Mitochondrial antioxidant expression in MS white matter

**Figure 1:**
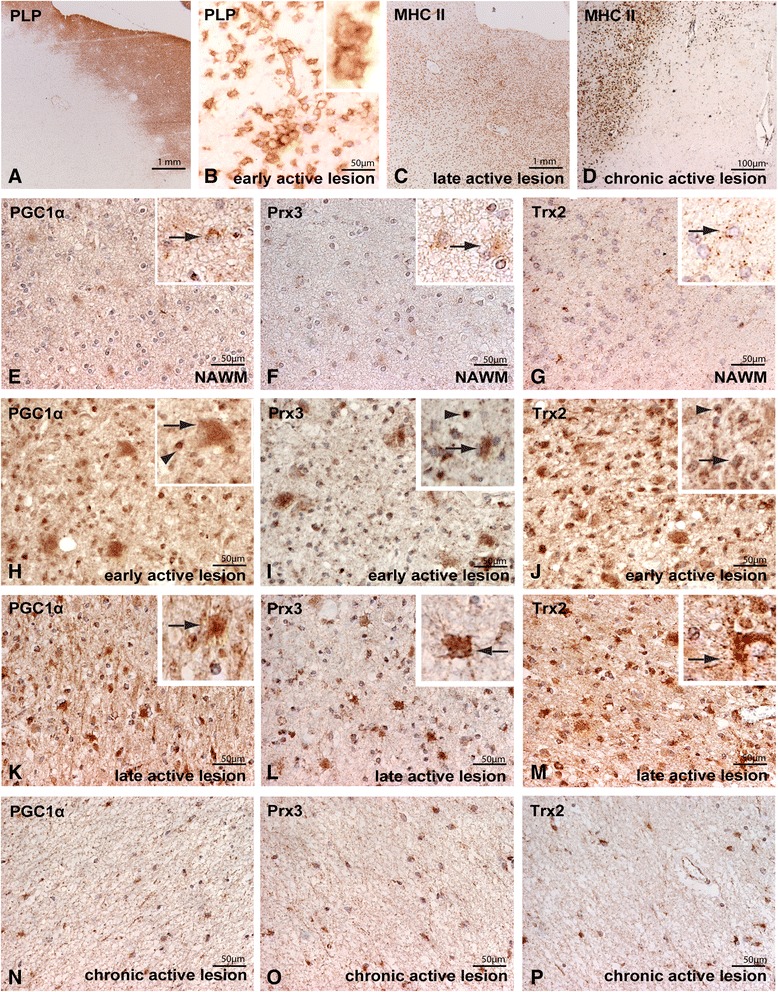
**Increased expression of PGC-1alpha and downstream mitochondrial antioxidants in active MS lesions.** MS lesions are characterized by loss of proteolipid protein (PLP; **A**). In early active lesions densely packed macrophages containing myelin proteins are present (**B**, inset), late active lesions typically contain MHCII positive leukocytes throughout the lesion area **(C)**. Chronic active MS lesions are characterized by a rim of activated microglia/macrophages at the border of the lesion which are absent in the lesion center **(D)**. Low PGC-1α, Prx3 and Trx2 immunostaining was observed in the NAWM (**E-G**, insets). Early active lesions showed enhanced PGC-1α, Prx3 and Trx2 immunoreactivity in astrocytes (arrow) and oligodendrocytes (arrowhead) (**H-J**, insets). In late active lesion expression is predominantly localized to astrocytes (arrow) (**K-M**, insets), which was largely lost in the inactive center of chronic active lesions **(N-P)**.

The early active lesion area in acute MS cases is characterized by severe demyelination (Figure 1A), massive microglial activation and the presence of densely packed macrophages containing myelin degradation products (Figure 1B) [33]. Late active MS lesions contain abundant leukocyte infiltrates throughout the lesion area, but lack PLP-positive macrophages (Figure 1C). Chronic active lesions are characterized by a rim of MHC class II-positive activated microglia and a demyelinated center devoid of inflammatory cells (Figure 1D).

**Figure 2:**
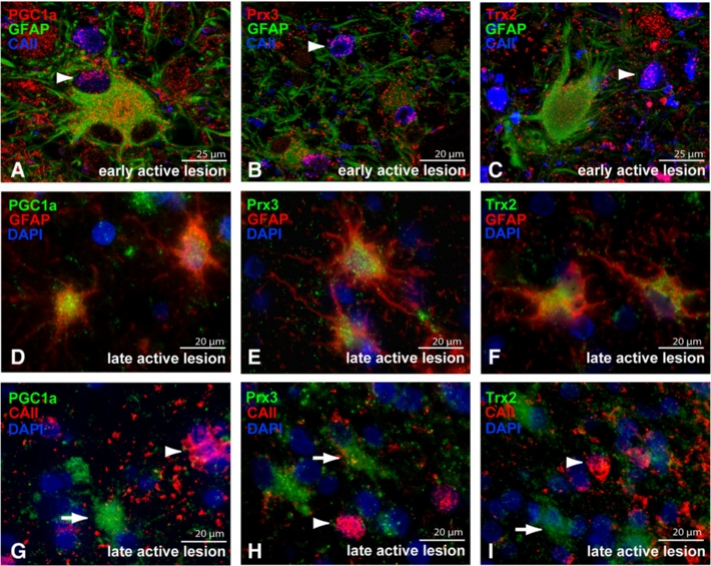
**PGC-1alpha, Prx3 and Trx2 are mainly expressed in astrocytes.** Triple immunofluorescent staining shows colocalization of PGC-1α (**A**, red), Prx3 (**B**, red), Trx2 (**C**, red) with GFAP-positive astrocytes (green) and CAII-positive oligodendrocytes (blue) in early active lesions. In late active lesions GFAP (red) positive cells colocalize with PGC-1α **(D)**, Prx3 **(E)** and Trx2 **(F)**. CAII positive oligodendrocytes (red, arrowheads) did not colocalize with PGC-1α **(G)**, Prx3 **(H)** and Trx2 **(I)** in late active lesions, whereas astrocyte like cells could be clearly distinguished (arrow).

PGC-1α, Prx3 and Trx2 were weakly expressed in astrocytes in normal appearing white matter (NAWM) (Figure 1E-G) and control white matter and no marked differences were observed comparing the intensity and cellular localization of PGC-1α, Prx3 and Trx2 immunostainings in NAWM with control white matter (data not shown). The expression of PGC-1α, Prx3 and Trx2 was strikingly upregulated in early active lesions compared to surrounding NAWM and localized to astrocytes (arrows) and oligodendrocytes (arrowheads) (Figure 1H-J). PGC-1α, Prx3 and Trx2 immunoreactivity was also consistently increased in late active lesions compared to NAWM, albeit less pronounced compared to early active lesions (Figure 1K-M). In late active lesions PGC-1α, Prx3 and Trx2 predominantly localized to cells with the morphological appearance of reactive astrocytes. In contrast to early active lesions, we did not find abundant oligodendrocyte expression of PGC-1α, Prx3 and Trx2 in late active lesions. Expression levels of PGC-1α, Prx3 and Trx2 in the inactive center of chronic active lesions were similar as observed in the NAWM (Figure 1N-P). Immunofluorescent triple stainings with glial fibrillary acidic protein (GFAP, astrocyte marker) and carbonic anhydrase II (CAII, oligodendrocyte marker) confirmed the cellular localization in astrocytes and oligodendrocytes (arrowhead) in early active lesions (Figure 2A-C). In late active MS lesions, PGC-1α (Figure 2D), Prx3 (Figure 2E) and Trx2 (Figure 2F) predominantly localized to astrocytes and to a much lesser extent to oligodendrocytes (2G-I). Mitochondrial antioxidant enzymes are weakly expressed in axons in active MS lesions (Additional file 3: Figure S1) and no apparent changes were observed between patients and controls (data not shown). Co-localization studies with the mitochondrial protein porin indicate that outside the nucleus, PGC-1α is expressed in mitochondria as described previously (Additional file 3: Figure S1) [34].

Taken together, the expression of PGC-1α and the mitochondrial antioxidant enzymes Prx3 and Trx2 is strongly increased in astrocytes in active MS lesions. In contrast, in the inactive center of chronic active demyelinated lesions the expression of PGC-1α, Prx3 and Trx2 returned to levels similar to the NAWM.

### Regulation and protective effect of astrocytic PGC-1α under oxidative stress

**Figure 3:**
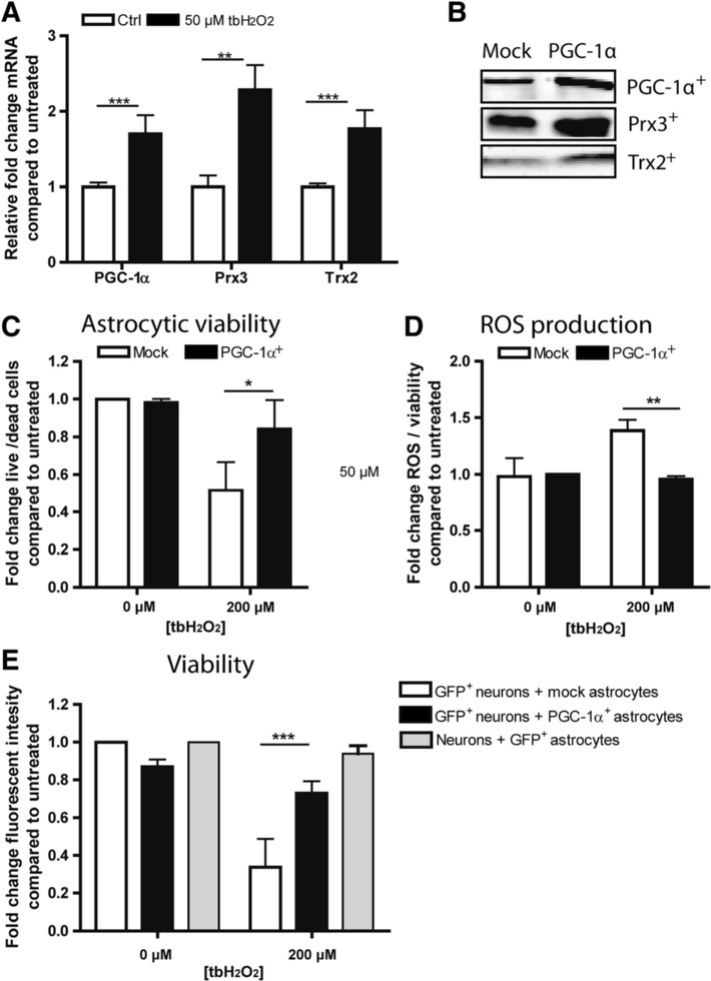
**Regulation and protective effects of astrocytic PGC-1alpha.** Treatment of human astrocytes with tbH_2_O_2_ increased mRNA expression levels of PGC-1α, Prx3 and Trx2 **(A)**. Western blot analysis illustrates that lentiviral overexpression of PGC-1α increases PGC-1α, Prx3 and Trx2 expression **(B)**. PGC-1α^+^ astrocytes were more resistant to tbH_2_O_2_ induced cell death **(C)** and produced less ROS compared to mock-transduced astrocytes **(D)**. SHSY5Y cells were better protected against tbH_2_O_2_ induced cell death when co-cultured with PGC-1α^+^overexpressing astrocytes compared to mock-transduced astrocytes **(E)**. GFP^+^ astrocytes co-cultured with SHSY5Y cells did not show reduced viability after tbH_2_O_2_ treatment **(E)**. *P < 0.05, **P < 0.01, ***P < 0.001 as determined by students T-test for figure **A**, **C**, **D** and with two-way ANOVA with post-hoc Bonferroni correction for Figure **E**.

To determine whether oxidative stress underlies the observed increase in astrocytic PGC-1α and mitochondrial antioxidant expression, human astrocytes were exposed to 50 μM tert-butyl hydrogen peroxide (tbH_2_O_2_) for 24 hours. Exposure to ROS significantly increased gene expression of Prx3, Trx2 and PGC-1α in human astrocytes (Figure 3A). Next, human astrocytes were stably transduced with PGC-1α containing lentivirus (PGC-1α^+^ astrocytes) thereby increasing expression of PGC-1α and its downstream targets Prx3 and Trx2 (Figure 3B). Overexpression of PGC-1α markedly increased the resistance of astrocytes against an oxidative attack (Figure 3C) and completely abolished oxidative stress-induced intracellular ROS production (Figure 3D). In order to assess the effect of PGC-1α upregulation in astrocytes on surrounding cells, GFP^+^ neuronal cells were co-cultured with mock-transduced or PGC-1α^+^ astrocytes and subsequently stimulated with 200 μM tbH_2_O_2_ for 6 hours. Neuronal cell death was significantly reduced when co-cultured with PGC-1α^+^ astrocytes compared to mock transduced astrocytes (Figure 3E). Importantly, the observed neuroprotective effect of PGC-1α^+^ astrocytes was not due to decreased astrocytic cell death in the PGC-1α^+^ astrocytes compared to mock astrocytes as GFP^+^ astrocytes co-cultured with GFP^−^ neuroblastoma cells showed virtually no cell death (Figure 3E).

As shown in Figure 3B, PGC-1α overexpression in astrocytes increased expression of Prx3 and Trx2, which likely contributes to the protective effect of PGC-1α against oxidative stress. Primary human astrocytes were only limited available, therefore we used U373 astrocytoma cells to assess whether Prx3 and Trx2 overexpression resulted in increased resistance against oxidative stress. Importantly, overexpression of PGC-1α in U373 cells showed similar protective effects as the PGC-1α^+^ astrocytes (Additional file 4: Figure S2). Overexpression of Prx3 and Trx2 in U373 astrocyte-like cells reduced ROS production and increased viability of astrocytes and surrounding neurons upon treatment with tbH_2_O_2_ (Additional file 4: Figure S2). Taken together, our data indicate that ROS induce upregulation of PGC-1α and downstream mitochondrial antioxidants, which in turn protects astrocytes and surrounding cells against ROS-mediated cell death.

### PGC-1α^+^ astrocytes have a reduced inflammatory profile

Reactive astrocytes in MS are known to produce high levels of the pro-inflammatory factors IL-6 and CCL2, which stimulate astrogliosis, increase vascular activation and enhance migration of leukocytes into the brain [35-37]. Previous studies have shown that increased PGC-1α expression can reduce the production of IL-6 and CCL2 in skeletal muscle cells [38,39]. This prompted us to investigate the role of PGC-1α on the expression of IL-6 and CCL2 in human astrocytes.

**Figure 4:**
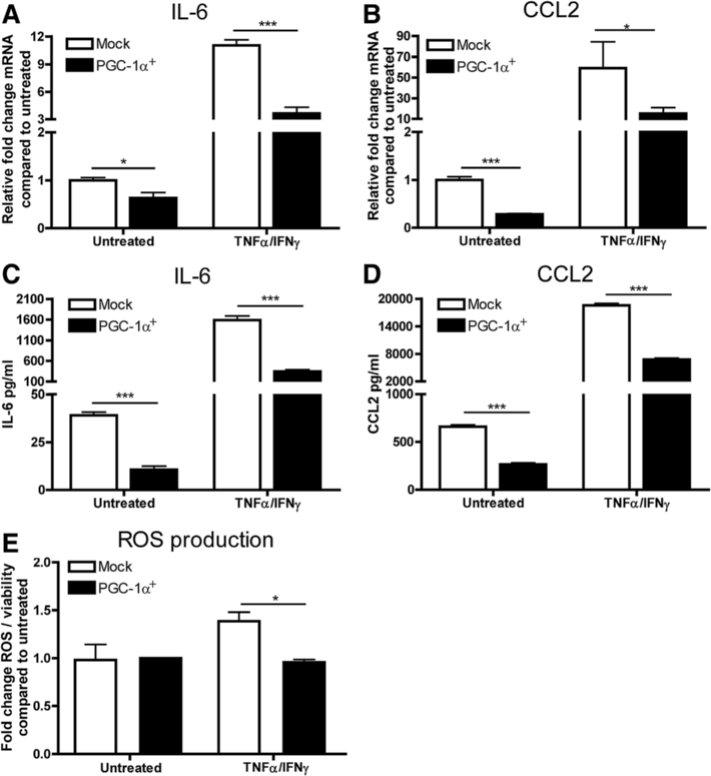
**Inflammatory profile of PGC-1alpha overexpressing astrocytes.** PGC-1α^+^ astrocytes expressed less IL-6 **(A)** and CCL2 **(B)** mRNA under normal conditions and after 24 hr treatment with TNFα/IFNγ compared to mock-transduced astrocytes. Secretion of IL-6 **(C)** and CCL2 **(D)** by astrocytes as measured by ELISA is reduced in PGC-1α^+^ astrocytes. TNFα/IFNγ increased ROS production in mock-transduced astrocytes but not in PGC-1α expressing astrocytes **(E)**. *P < 0.05, **P < 0.01, ***P < 0.001 as determined by students T-test.

TNF-α and IFN-γ are present in high concentrations in inflammatory MS lesions and treatment of astrocytes with TNF-α and IFN-γ is a useful tool to create reactive astrocytes *in vitro* [7,40,41]. Interestingly, our results show that PGC-1α^+^ astrocytes express the pro-inflammatory genes IL-6 and CCL2 at lower levels under basal conditions and upon stimulation with TNF-α/IFN-γ compared to mock transduced astrocytes (Figure 4A-B). Secretion of IL-6 and CCL2 was also reduced in PGC-1α^+^ astrocytes (Figure 4C-D). Next, we investigated whether PGC-1α overexpression decreased cytokine-induced ROS production. Notably, TNF-α/IFN-γ-mediated ROS production was significantly reduced in PGC-1α^+^ astrocytes compared to mock transduced astrocytes (Figure 4E). Our findings indicate that PGC-1α, besides controlling mitochondrial redox metabolism, exerts a profound effect on the inflammatory profile of astrocytes.

## Discussion

In the present study we show that the expression of the transcription co-factor proliferator-activated receptor gamma coactivator 1-alpha (PGC-1α) and downstream mitochondrial antioxidant enzymes peroxiredoxin-3 (Prx3) and thioredoxin-2 (Trx2) is markedly increased in astrocytes in active multiple sclerosis (MS) lesions. Our *in vitro* data provide evidence that overexpression of PGC-1α protects human astrocytes against oxidative stress and reduces intracellular ROS production. Moreover, co-culture experiments indicate that increased expression of PGC-1α in astrocytes protects neurons from ROS-induced cell death. Finally, we demonstrate that PGC-1α overexpression reduces the production of astrocyte-derived inflammatory molecules.

ROS unambiguously play a cardinal role in MS pathology as recent studies demonstrate a clear association between inflammation-derived ROS, mitochondrial (dys)function and neurodegeneration [12]. Since little is known about the distribution and functional role of mitochondrial enzymes, we set out to investigate the expression of PGC1-α and its downstream targets Prx3 and Trx2 in a large selection of white matter MS lesions. In early active MS lesions, which represent the initial phase of MS lesions, we found a striking increase in the expression of PGC1-α, Prx3 and Trx2 in both astrocytes and oligodendrocytes compared to NAWM and control tissue [42]. Our *in vitro* experiments demonstrated that astrocytes strongly upregulate PGC1-α and mitochondrial antioxidants upon exposure to ROS, which is in line with previous studies describing ROS-induced PGC1-α expression in mouse muscle and embryonic mesenchymal stem cells [43,44]. This likely represents a protective response to the local oxidative milieu. In fact, we show that overexpression of PGC-1α or mitochondrial antioxidants protects astrocytes from ROS-induced cell death, which corroborates previous studies using different cell types and experimental animal models [21,45-47]. In late active MS lesions, enhanced PGC1-α, Prx3 and Trx2 immunoreactivity was mainly observed in astrocytes, not oligodendrocytes. This finding suggests that surviving oligodendrocytes may lose their ability to express adequate levels of mitochondrial antioxidants in time, making them more vulnerable to ROS-induced cell death. Notably, oligodendrocyte loss is a key feature of inflammatory MS lesions, whereas astrocytes generally survive. Our findings are in line with previous data from our group in which we showed that astrocytes effectively induce cytoplasmic antioxidant levels in late active MS lesions [10]. Besides oligodendrocytes, axons represent the main victims of the oxidative attack in inflammatory lesions [25]. Remarkably, similar to previous observations for cytoplasmic antioxidants, we found no evident increase in mitochondrial antioxidant defence mechanisms in axons in any stage of MS pathology [10]. A likely explanation is that the distance between the cell body, where PGC-1α induces expression of mitochondrial antioxidants, and the affected axon is too large for a timely and efficient antioxidative response. Moreover, we previously showed that PGC-1α, Prx3 and Trx2 are decreased in cortical neurons of MS patients, suggesting that reduced PGC-1α in cortical neuronal cell bodies might contribute to the lack of mitochondrial antioxidant induction in demyelinated axons [22]. Taken together, the relative inability of axons and oligodendrocytes to increase their mitochondrial antioxidative capacity in response to an oxidative attack likely contributes to the extensive oxidative damage of axons and oligodendrocytes and subsequent cell death in MS lesions.

Loss of PGC-1α in cultured neurons induces intracellular ROS production and the susceptibility to ROS-induced cell death [22]. In this study we show for the first time that enhanced astrocytic expression of PGC-1α and mitochondrial antioxidants is able to protect adjacent neurons from exogenous ROS *in vitro*. A potential explanation for the intriguing neuroprotective effect is that astrocytes can shuttle antioxidants to neurons, thereby enhancing neuronal antioxidant capacity. This has been shown for glutathione, but it remains unknown whether Prx3 and Trx2 are also secreted by astrocytes [48,49]. Interestingly, PGC-1α can also stimulate the production of enzymes involved in glutathione biosynthesis [50]. However, we demonstrate that overexpression of astroglial Prx3 or Trx2 alone is sufficient to protect surrounding neurons against an oxidative insult. The precise mechanism how enhanced levels of astroglial PGC-1α and downstream mitochondrial antioxidant enzymes protect neighbouring cells warrants future research, but astrocytes may scavenge extracellular ROS and by preserving their mitochondrial function during oxidative stress, astrocytes remain able to provide support to adjacent neurons. Nonetheless, neuro-axonal damage is profound in (early) active MS lesions indicating that the increase in astroglial mitochondrial antioxidants is not sufficient to protect all neighbouring axonal structures and cells [25].

PGC-1α regulates the transcription of a broad array of genes and is a key player in mitochondrial biogenesis, consequently it is conceivable that the protective effects of enhanced PGC-1α levels extend beyond increased mitochondrial antioxidant production [24]. Since PGC-1α has such widespread effects and evidence for an intricate relationship between cellular metabolism and inflammation is mounting, we were interested whether astroglial PGC-1α was able to influence the production of inflammatory mediators [51,52]. In inflammatory MS lesions, reactive astrocytes are known to produce high amounts of IL-6 and CCL2 thereby contributing to the ongoing inflammation [35,36]. Interestingly, PGC-1α overexpression strongly reduced the production of astrocytic IL-6, CCL2 and ROS upon treatment with key pro-inflammatory mediators. The anti-inflammatory effects of PGC-1α could be mediated by PGC-1α-induced PPAR signalling which is known to reduce IL-6 and CCL2 production by astrocytes, however future studies are needed to elucidate the anti-inflammatory properties of PGC-1α [53].

This study provides novel insights in the protective properties of reactive astrocytes. We previously showed that reactive astrocytes produce both pro- and anti-inflammatory mediators, but they are vital for tissue repair since ablation of astrocyte activation enhances CNS damage in various experimental models [6,7,54-56]. Thus, therapies aimed at augmenting the production and activity of mitochondrial antioxidants in neurons, but also in astrocytes, represent an interesting strategy to restore mitochondrial function and combat neurodegeneration. Activation of the PGC-1α pathway is of particular interest since this will not only boost the (mitochondrial) antioxidants machinery, but, in addition, also promotes expression of a variety of proteins involved in energy metabolism and suppresses inflammation [26]. Several studies have shown that resveratrol and progesterone, both known to activate PGC-1α, are neuroprotective and reduce clinical symptoms in experimental autoimmune encephalitis (EAE) [57-59]. Interestingly, overexpression of sirtuin 1 (Sirt1), a direct activator of PGC-1α, is also neuroprotective and reduces clinical symptoms in EAE [60].

## Conclusion

**Figure 5:**
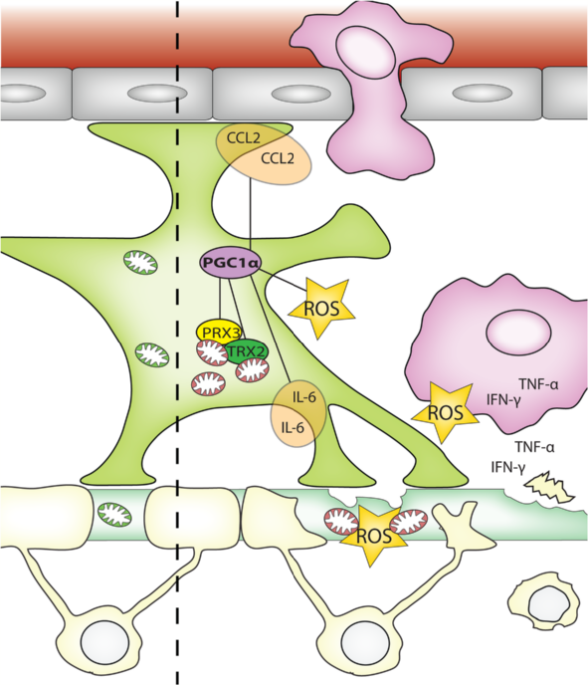
**Graphical abstract.** Macrophages are attracted by CCL2 and infiltrate the brain, phagocytose myelin and produce high amounts of ROS and TNF-α and IFN-γ. This will lead to mitochondrial stress, resulting in more mitochondrial ROS production contributing to axonal degeneration. Increased amounts of ROS induce astrocytic PGC-1α, which increases mitochondrial antioxidant capacity via upregulation of Prx3 and Trx2. Increased expression of PGC-1α, Prx3 or Trx2 protects astrocytes and surrounding neurons not only from mitochondrial stress but also against exogenous ROS. Interestingly, PGC-1α can reduce CCL2 and IL-6 production and TNF-α/IFN-γ induced ROS production, thereby dampening inflammation. Thus increased astrocytic PGC-1alpha can reduce ROS and inflammation in MS lesions which protects surrounding axons and neurons.

In conclusion, we have shown increased PGC-1α and downstream mitochondrial antioxidant enzyme expression in astrocytes in inflammatory MS lesions. Overexpression of PGC-1α in primary human astrocytes attenuates intracellular ROS production and protects astrocytes as well as surrounding cells from ROS-mediated cell death. Moreover, we provide evidence that PGC-1α limits the production of astrocyte-derived inflammatory molecules (Figure 5). Hence, activation of the PGC-1α pathway represents an attractive approach to limit both inflammation and oxidative stress, two key features of MS pathogenesis.

## Additional files

Additional file 1: Table S1. Antibody details. Additional file 2: Table S2. Primer sequences. Additional file 3: Figure S1. Prx3 (**A**, **C**, green) and Trx2 (**B**,**D**, green) staining was found to colocalize with both non-phosphorylated- (SMI32; in red) and phosphorylated axons (SMI31, in red). PGC-1α is localized in both mitochondria (porin, red) and nuclei (DAPI, blue) (N). Additional file 4: Figure S2. Western blot analysis shows that U373 astrocyte-like cells treated with Prx3 and Trx2 containing lentiviral constructs have increased Prx3 and Trx2 protein expression compared to mock transduced U373 cells **(A)**. PGC-1α^+^ U373 have increased protein expression of PGC-1α, Prx3 and Trx2 **(B)**. Live/dead viability assay revealed reduced vulnerability of astrocyte-like cells overexpressing Prx3, Trx2 or PGC-1α to tbH_2_O_2_ treatment **(C,D)**. ROS production was increased in mock-transduced U373s compared to Prx3^+^, Trx2^+^ or PGC-1α^+^ U373 cells **(E,F)**. GFP^+^ SH5YSY cells were better protected against tbH_2_O_2_ when cultured together with Prx3^+^, Trx2^+^ or PGC-1α^+^ astrocytes compared to mock-transduced astrocytes **(G,H)**. U373 cells treated with the same concentrations of tbH_2_O_2_ as the neuronal cultures showed no difference in viability between the different cell lines **(I,J)**. Significance was compared to Mock cells. *P < 0.05, **P < 0.01, ***P < 0.001 as determined by two-way ANOVA with post-hoc Bonferroni correction.
