# Supplementary material for: Astroglial PGC-1alpha increases mitochondrial antioxidant capacity and suppresses inflammation: implications for multiple sclerosis
Source: Acta Neuropathol Commun. 2014 Dec 10;2:170. doi: 10.1186/s40478-014-0170-2 (PMC4268800; doi:10.1186/s40478-014-0170-2)
Supplement: Additional file 1: Table S1. — Antibody details. [file 40478_2014_170_MOESM1_ESM.doc]

| **Supplementary table 1.** Antibody details | | | | |
| --- | --- | --- | --- | --- |
| **Antigen** | **Dilution** | | **Antibody type** | **Source*** |
|  | **IHC** | **WB** |  |  |
| Proteolipid protein (PLP) | 1:500 | NA | IgG2a | Serotec |
| HLA-DR | 1:50 | NA | IgG2b | eBioscience |
| PGC-1α | 1:100 | 1:1000 | rabbit polyclonal | Santa Cruz |
| Peroxiredoxin (Prx)3 | 1:15000 | 1:5000 | rabbit polyclonal | Abfrontier |
| Thioredoxin (Tnx)2 | 1:100 | 1:500 | rabbit polyclonal | Sigma |
| GFAP | 1:10 | NA | IgG | Monosan |
| Actin | NA | 1:1000 | goat polyclonal | Santa Cruz |
| Carbonic anhydrase (CAII) | 1:500 | NA | sheep polyclonal | Binding site |
| Neurofilaments, phosphorylated (Smi 31) | 1:1000 | NA | IgG1 | Covance |
| Neurofilament H, non-phosphorylated (Smi 32) | 1:100 | NA | gG1 | Covance |
| IHC, immunohistochemistry; WB, western blot; HLA-DR, human leukocyte antigen-DR; PGC-1α, peroxisome proliferator-activated receptor-γ coactivator-1-α; GFAP, glial fibrillary acidic protein; NA, not applicable  * Sources: Serotec, Oxford, UK; eBioscience, San Diego, CA; Santa Cruz Biotechnology, Santa Cruz, CA; Abfrontier, Seoul, Korea; Sigma-Aldrich, St Louis, MO; Monosan, Uden, The Netherlands; Binding site, Birmingham, UK; Covance, Emeryville, CA. | | | | |
