# Supplementary material for: Astroglial PGC-1alpha increases mitochondrial antioxidant capacity and suppresses inflammation: implications for multiple sclerosis
Source: Acta Neuropathol Commun. 2014 Dec 10;2:170. doi: 10.1186/s40478-014-0170-2 (PMC4268800; doi:10.1186/s40478-014-0170-2)
Supplement: Additional file 2: Table S2. — Primer sequences. [file 40478_2014_170_MOESM2_ESM.doc]

**Supplementary Table 2. Primer sequences**

| **Target gene** | **Forward primer** | **Reverse primer** |
| --- | --- | --- |
| PGC-1α | ACAGCCGTCGGCCCAGGTAT | GCCTCTCCCTTTGCTTGGCCC |
| Prx3 | CTGGATAAATACACCAAGGAAGA | CTTCTAACAGCACACCGT |
| Trx2 | GGATTTCCACGCACAGT | CCGCTGACACCTCATACT |
| IL-6 | GGTACATCCTCGACGGCATCT | GTGCCTCTTTGCTGCTTTCAC |
| CCL2 | CCCTTCTGTGCCTGCTGCTCA | CTGTTCGTTTGGGTTTGAGGCTT |
