# Supplementary figures and images for: Astroglial PGC-1alpha increases mitochondrial antioxidant capacity and suppresses inflammation: implications for multiple sclerosis
Source: Acta Neuropathol Commun. 2014 Dec 10;2:170. doi: 10.1186/s40478-014-0170-2 (PMC4268800; doi:10.1186/s40478-014-0170-2)

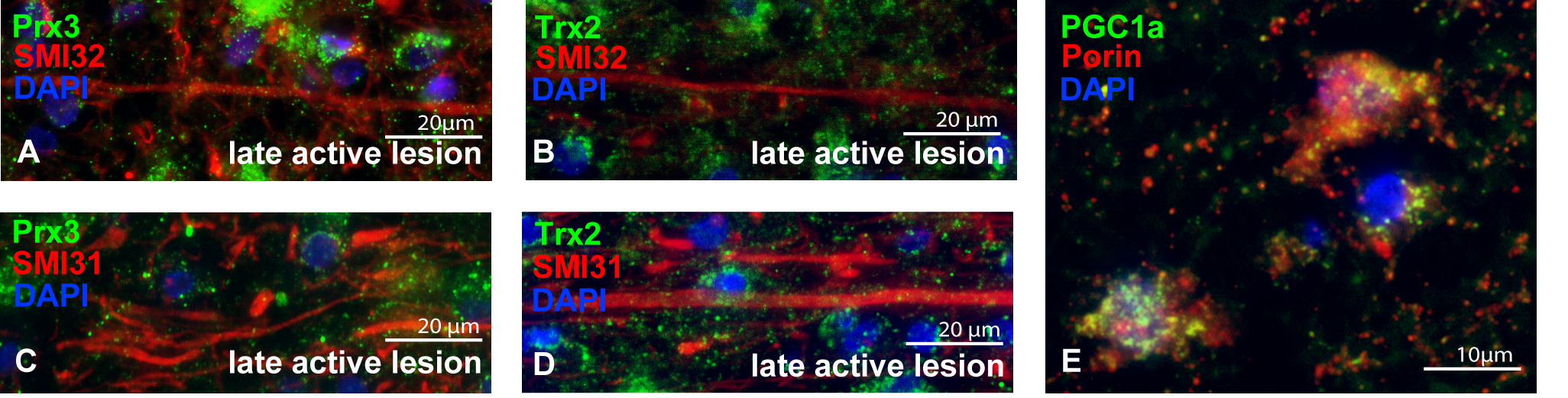

Supplement: Additional file 3: Figure S1. — Prx3 (A, C, green) and Trx2 (B,D, green) staining was found to colocalize with both non-phosphorylated- (SMI32; in red) and phosphorylated axons (SMI31, in red). PGC-1α is localized in both mitochondria (porin, red) and nuclei (DAPI, blue) (N). [file 40478_2014_170_MOESM3_ESM.tif]

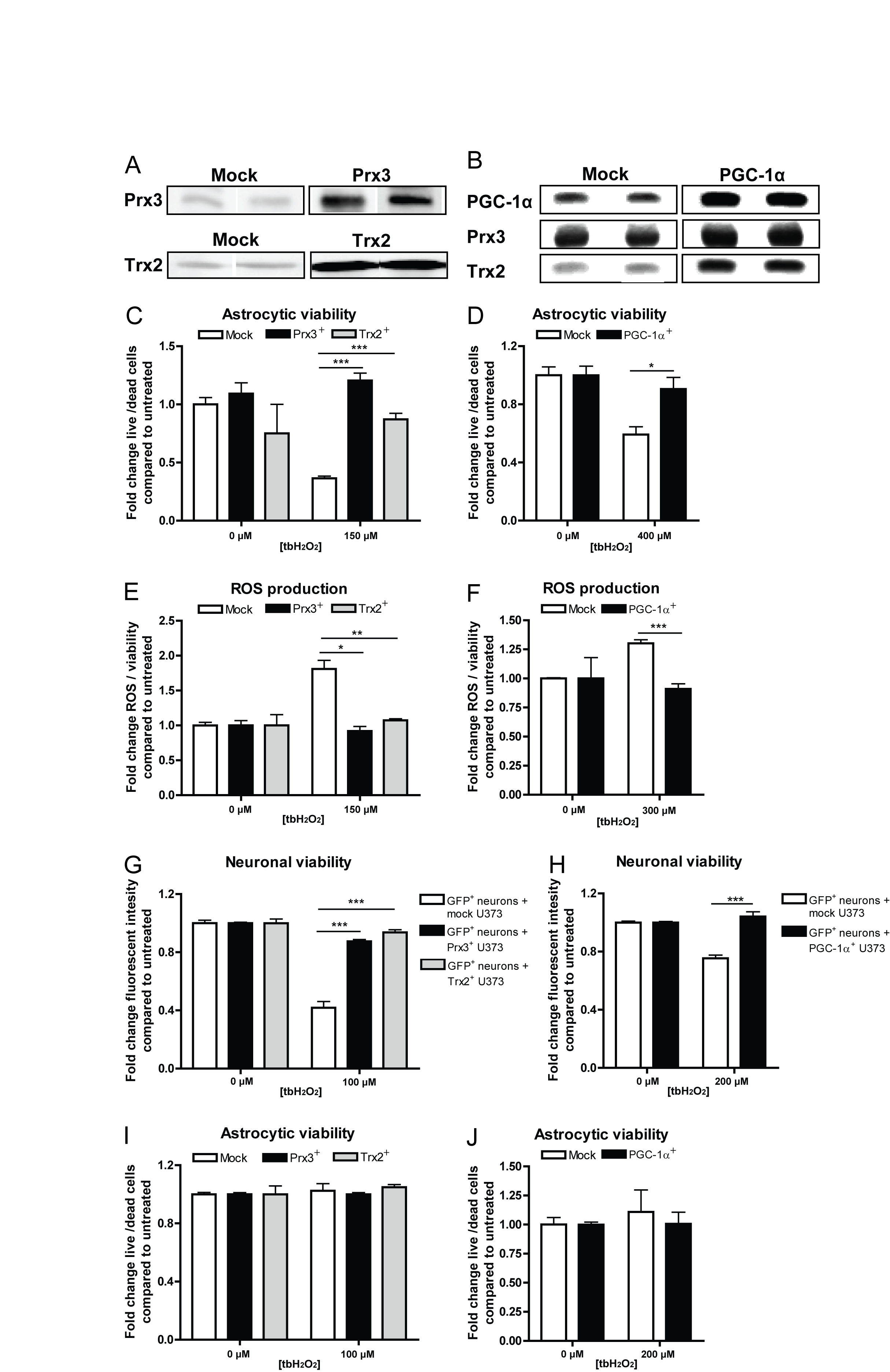

Supplement: Additional file 4: Figure S2. — Western blot analysis shows that U373 astrocyte-like cells treated with Prx3 and Trx2 containing lentiviral constructs have increased Prx3 and Trx2 protein expression compared to mock transduced U373 cells (A). PGC-1α+ U373 have increased protein expression of PGC-1α, Prx3 and Trx2 (B). Live/dead viability assay revealed reduced vulnerability of astrocyte-like cells overexpressing Prx3, Trx2 or PGC-1α to tbH2O2 treatment (C,D). ROS production was increased in mock-transduced U373s compared to Prx3+, Trx2+ or PGC-1α+ U373 cells (E,F). GFP+ SH5YSY cells were better protected against tbH2O2 when cultured together with Prx3+, Trx2+ or PGC-1α+ astrocytes compared to mock-transduced astrocytes (G,H). U373 cells treated with the same concentrations of tbH2O2 as the neuronal cultures showed no difference in viability between the different cell lines (I,J). Significance was compared to Mock cells. *P < 0.05, **P < 0.01, ***P < 0.001 as determined by two-way ANOVA with post-hoc Bonferroni correction. [file 40478_2014_170_MOESM4_ESM.tif]
